# Author Correction: Atypical function of a centrosomal module in WNT signalling drives contextual cancer cell motility

**DOI:** 10.1038/s41467-025-57127-8

**Published:** 2025-02-26

**Authors:** Yi Luo, Miriam Barrios-Rodiles, Gagan D. Gupta, Ying Y. Zhang, Abiodun A. Ogunjimi, Mikhail Bashkurov, Johnny M. Tkach, Ainsley Q. Underhill, Liang Zhang, Mohamed Bourmoum, Jeffrey L. Wrana, Laurence Pelletier

**Affiliations:** 1 Lunenfeld-Tanenbaum Research Institute, Mount Sinai Hospital, Toronto, ON M5G 1X5 Canada; 2 Department of Molecular Genetics, University of Toronto, Toronto, ON M5S 1A8 Canada; 3 Present Address: Department of Chemistry and Biology, Ryerson University, Toronto, ON M5B 2K3 Canada; 4 Present Address: Department of Biomedical Sciences, City University of Hong Kong, Hong Kong, China

Correction to: *Nature Communications* 10.1038/s41467-019-10241-w, published online 29 May 2019

In the version of the article initially published, in Fig. 9g, the image for DAAM2 (ROI), DMEM (DAAM1) was a duplicate of the image above it (DAAM2 (ROI), ACM (Ctrl)) and has now been replaced in the HTML and PDF versions of the article.

