# Supplementary material for: Atypical function of a centrosomal module in WNT signalling drives contextual cancer cell motility
Source: Nat Commun. 2019 May 29;10:2356. doi: 10.1038/s41467-019-10241-w (PMC6541620; doi:10.1038/s41467-019-10241-w)
Supplement: Supplementary file 1 — Supplementary Information [file 41467_2019_10241_MOESM1_ESM.pdf]

Supplementary Information

Atypical Function of a Centrosomal Module in WNT Signalling Drives Contextual Cancer Cell Motility

Luo et al.

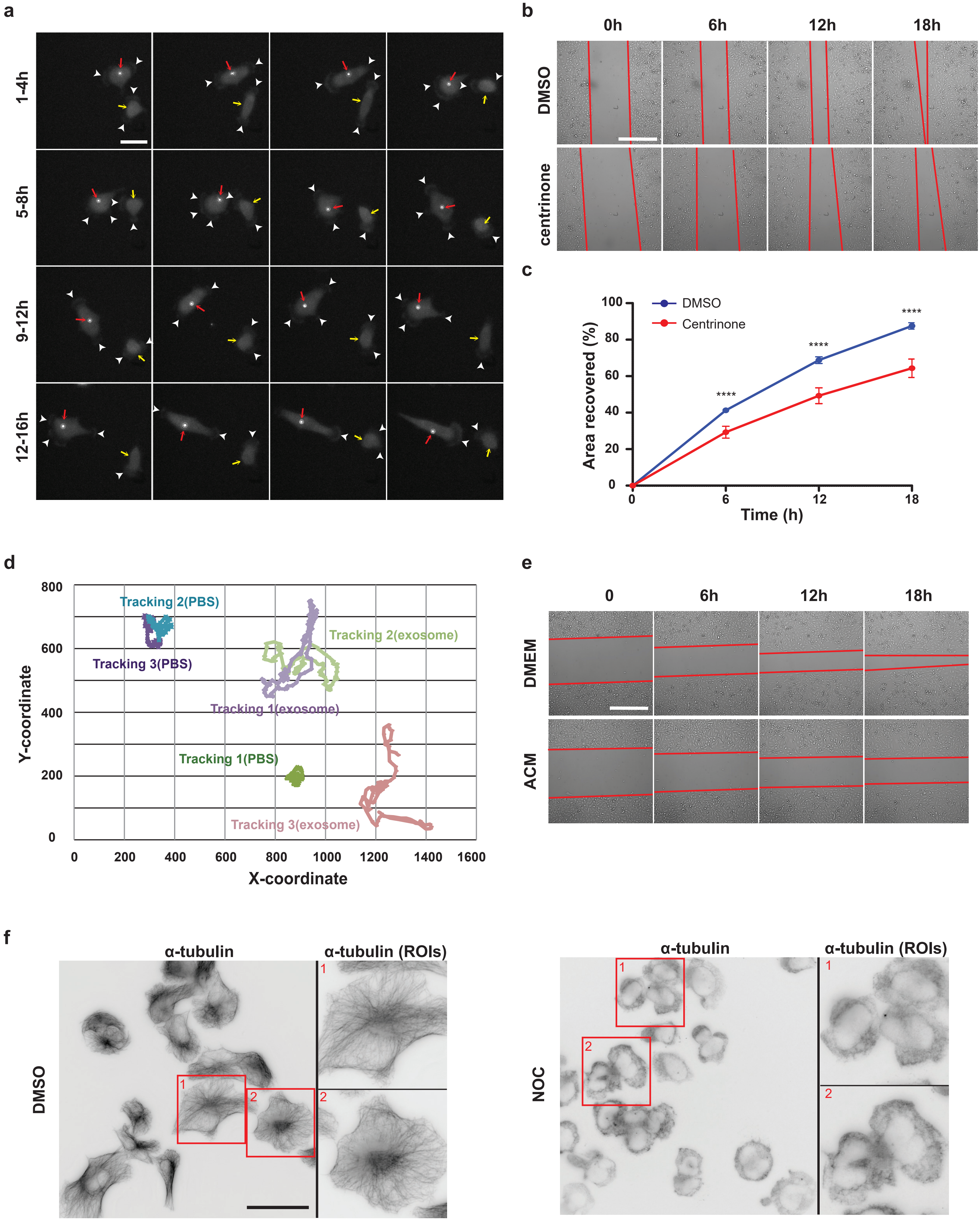

Luo et al., Supplementary Figure 1

Supplementary Figure 1. Exosome-induced BCC motility is centrosome- and MT-independent. a, MDA-MB-231 cells stably expressing GFP-CETN2 were treated with DMSO or centrinone for 5 days. At the end of incubation, cells treated with DMSO (centrosome present) and centrinone (centrosome depleted) were mixed at 1:1 ratio, and were stimulated with ACM for 16h. Time-lapse images were then taken as described in Methods. Montage of fluorescence images show the morphology of a control cell (red arrow) and a centrosome-depleted cell (yellow arrow) at every hour. Cell protrusions are indicated by arrowheads. **b, c,** Representative images and quantitative data of wound healing assay on cells treated with DMSO or centrinone for 5 days. Graph in **c** shows mean area recovered ± s.e.m. Data are compared with Two-Way ANOVA post-tested with Bonferroni test (****$p<0.0001$; N=3, at least 30 regions were measured per condition). **d,** Cell tracking traces in an XY Coordinates map. Migrating traces of three cells from each group treated with either PBS or purified exosomes are shown as examples (N=3). **e,** Representative images of wound healing assay on MDA-MB-231 cells treated overnight with DMEM or ACM. **f,** MDA-MB-231 cells treated with DMSO or NOC for 2h and stained for immunofluorescence with α-tubulin to observe the microtubule network. Enlarged regions of interest (ROIs) are shown on the right. Bar=50 µm. (N=3).

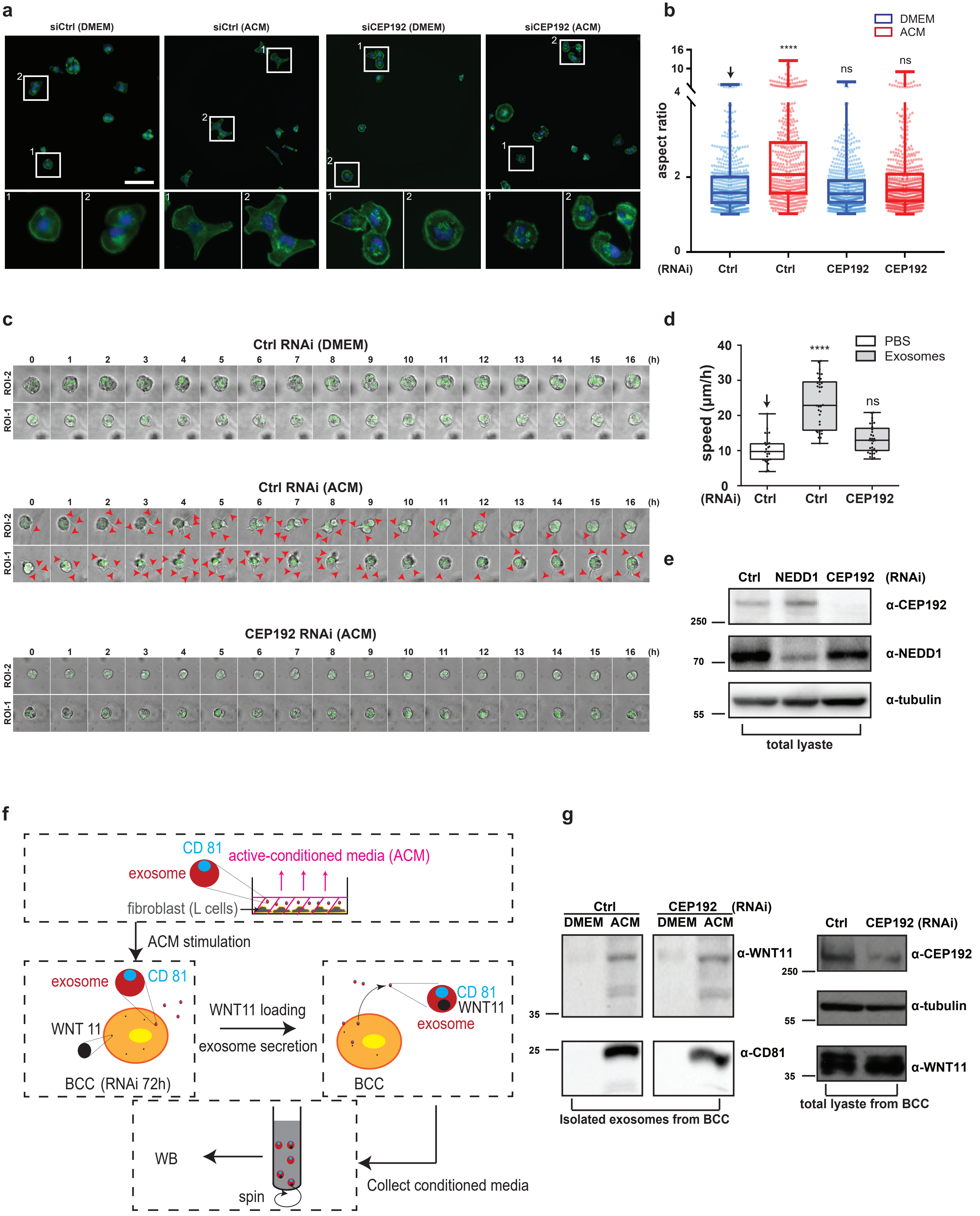

Luo et al., Supplementary Figure 2

Supplementary Figure 2. CEP192 depletion disrupts exosome-induced cancer cell motility but not WNT11 trafficking. a, MDA-MB-231 cells were treated with the indicated siRNAs for 72h and then incubated with DMEM or ACM overnight. Cell was labeled with DAPI (blue) and actin (green). Enlarged box areas (ROIs) are shown in lower panels. Bar=50μm. (N=3). **b,** Cell aspect ratio quantification in cells from (**a**) using MATLAB. Aspect ratio =1 indicates a perfectly round shape. (**** p<0. 0001; N=3, at least 200 cells were measured per condition). **c,** MDA-MB-231 cells stably expressing GFP-tagged Histone 2B (GFP-H2B) were transfected with control or CEP192 siRNA 48h before embedding into Matrigel as described in Methods and further incubated for 24h in the presence of DMEM or ACM. Live imaging was captured every 15min for 16h from both bright field and GFP as described in Methods. Montage images show the morphology (bright field) and nucleus (GFP) of spheroids (about 4-6 cells size) at every hour from boxes highlighted in **Supplementary Movie 3**. Cell protrusions are indicated by red arrowheads. **d,** MDA-MB-231 cells were transfected with control or CEP192 siRNAs for 72h. Cells were then stimulated with either PBS or purified exosomes resuspended in PBS, and cell motility was measured as described in Methods. (****p<0. 0001; N=3, at least 30 cells were tracked per condition). **e,** MDA-MB-231 cells treated with control, NEDD1 or CEP192 siRNAs for 72h and then lysed. Western blotting was performed using the indicated antibodies to confirm knockdown efficiency. (N=3). **f,** Diagram of experimental design to determine CD81-positive exosomes loaded with endogenous Wnt11 and then released by BCCs. MDA-MB-231 cells were treated with siRNAs as indicated for 72h, followed by overnight incubation with control media or ACM (containing CD81-positive exosomes from L cells). The conditioned media released by MDA-MB-231 cells was then collected and centrifuged to purify exosomes as described in Methods. Exosomes were resuspended in sample buffer, and then western bolt was performed. **g,** MDA-MB-231 cells were treated with control or CEP192 siRNA for 72h and then stimulated with control media or ACM as illustrated in (**e**). Wnt 11 and CD 81 levels in purified exosomes released by MDA-MB-231 (left side panels), and CEP192, α-tubulin and Wnt11 in total lysates (right side panel) were detected by western blotting. All data were plotted as box and whiskers. Boxes represent median and 25th to 75th percentiles, whiskers the minimum and maximum values with each individual cell value superimposed. Data were compared with one-way ANOVA Kruskal-Wallis Test and post-tested with Dunn's Multiple Comparison Test. Arrow indicates the control bar used for comparison.

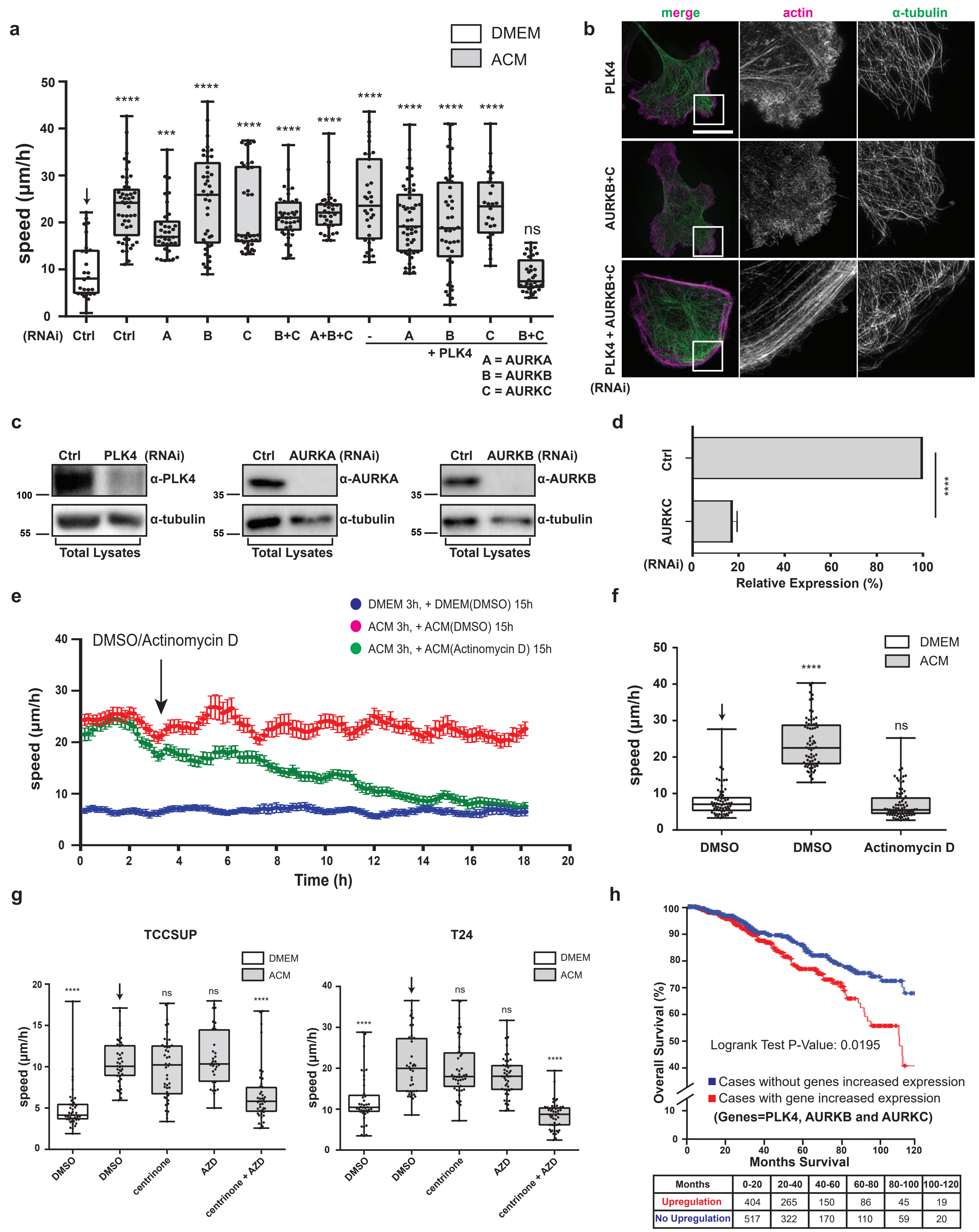

Months	0-20	20-40	40-60	60-80	80-100	100-120
Upregulation	404	265	150	86	45	19
No Upregulation	517	322	170	110	59	20

(Total Patient Number Involved In Program)

Luo et al., Supplementary Figure 3

i

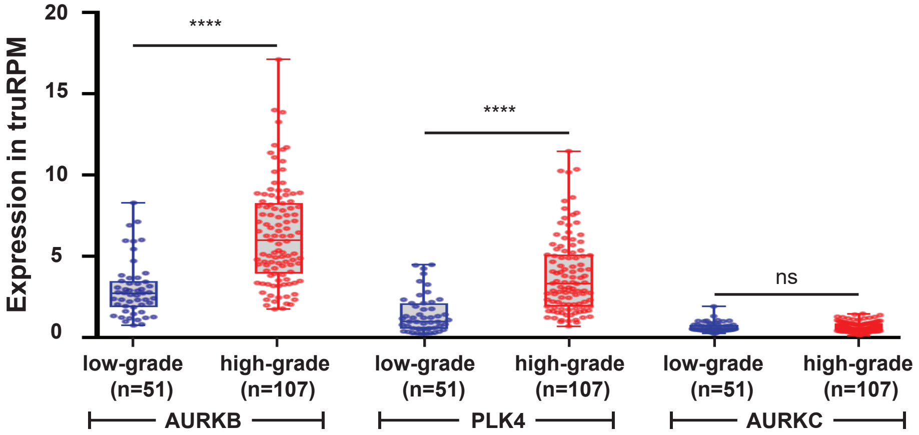

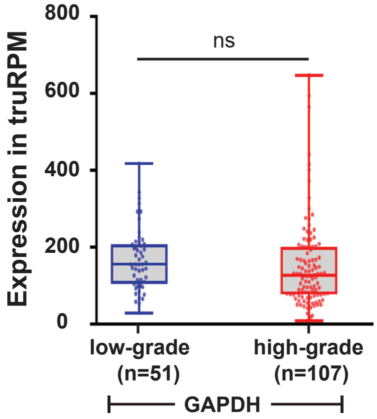

Supplementary Figure 3. PLK4 and AURKB increased expression correlate with poor cancer prognosis and survival. a, MDA-MB-231 cells were transfected with siRNAs as indicated for 72h. After RNAi treatment, cells were stimulated with DMEM or ACM and cell motility was measured. (***p<0. 001, ****p<0. 0001; N=3, at least 50 cells were tracked per condition). **b,** Representative images of cells from (**a**) were stained for α-tubulin and actin. Enlarged areas on the right side panels show cortical regions. Bar=20 μm. (N=3). **c,** Lysates from MDA-MB-231 cells treated with siRNAs for 72h were analyzed by western blotting to confirm knockdown using specific antibodies. **d,** AURKC mRNA expression levels in MDA-MB-231 cells were determined by RT-qPCR in cells transfected for 72h with control or AURKC siRNA used in (**a**). Bar graph shows average ± s.e.m of 4 independent experiments (****p<0. 0001). **e,** MDA-MB-231 cells were stimulated with DMEM or ACM overnight. Cell motility was tracked for 3h, then cells were treated with DMSO or Actinomycin D and further tracked for 16h. Running average speed is plotted as mean ± s.e.m (N=3, at least 40 cells were tracked per condition). **f,** MDA-MB-231 Cells were stimulated with DMEM or ACM for 16 h in the presence of DMSO or 0.5μg/ml Actinomycin D. Cell motility was measured as described in Methods. (****p<0.0001; N=3, at least 40 cells were tracked per condition). **g,** Bladder cancer cell lines, TCCSUP and T24 were stimulated with DMEM or ACM in the presence of DMSO or inhibitors as indicated. Cell motility was tracked as described in Methods.(****p<0. 0001; N=3, at least 40 cells were tracked per condition). **h,** Survival data of breast invasive carcinoma patients with or without PLK4, AURKB and/or AURKC increased expression that includes mRNA expression upregulation (RNA seq V2 R s.e.m, z-score ≥1.7), DNA copy-number amplification and gain (top panel). Total number of patients involved in the program at each time period is shown (bottom table). Graph is based on data from TCGA Research Network[68,69] (TCGA Provisional, http://www.cbioportal.org). **i,** RNA expression levels for PLK4, AURKB, AURKC and GAPDH in low grade and high-grade bladder cancer patient samples analyzed by RNA-seq as described in Methods (**** p<0. 0001). Number of patients from each group are indicated in X axis. Data from **d** and **i** wereanalyzed by Mann-Whitney U test Two-tailed. Data from **a, f, g** and **i** were plotted as box and whiskers. Boxes represent median and 25th to 75th percentiles, whiskers the minimum and maximum values with each individual cell value superimposed. Data from **a, f** and **g** were compared with one-way ANOVA Kruskal-Wallis Test and post-tested with Dunn's Multiple Comparison Test. Arrow indicates the control bar used for comparison.

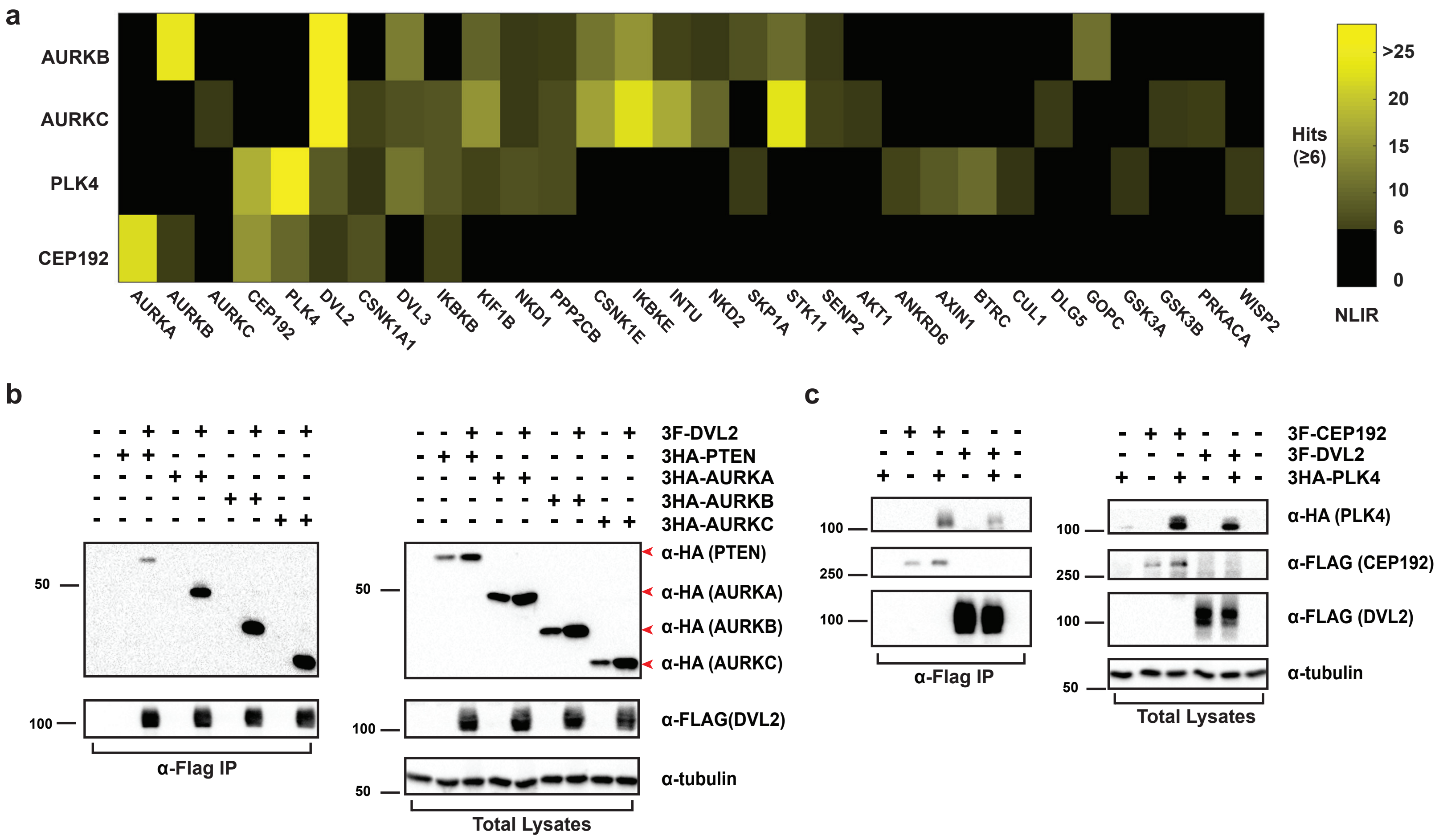

Supplementary Figure 4. DVL2 binds to CEP192, PLK4 and AURKB. a, Heatmap of the average results from two independent LUMIER screens using AURKB, AURKC, PLK4 and CEP192 as baits tested against the indicated 3Flag-tagged preys. A NLIR ≥6[59,60] was used as a threshold to define the hits shown in the heatmap. All bait-prey pairs tested are listed in Supplementary Table 1. **b,** HEK293T cells were co-transfected with 3Flag-DVL2 and 3HA-AURKs or 3HA-PTEN (positive control)[56] as indicated. Cell lysates were immunoprecipitated with anti-Flag antibody and AURKs or PTEN were detected by western blotting using anti-HA antibody. Red arrowheads indicate bands corresponding to 3HA-AURKs or 3H-PTEN. (N=3). **c,** HEK293T cells were co-transfected with 3Flag-DVL2 or 3Flag-CEP192 (positive control)[56] and 3HA-PLK4 as indicated. Cell lysates were immunoprecipitated with anti-Flag antibody and 3HA-PLK4 was detected by western blotting as in (**c**). (N=3).

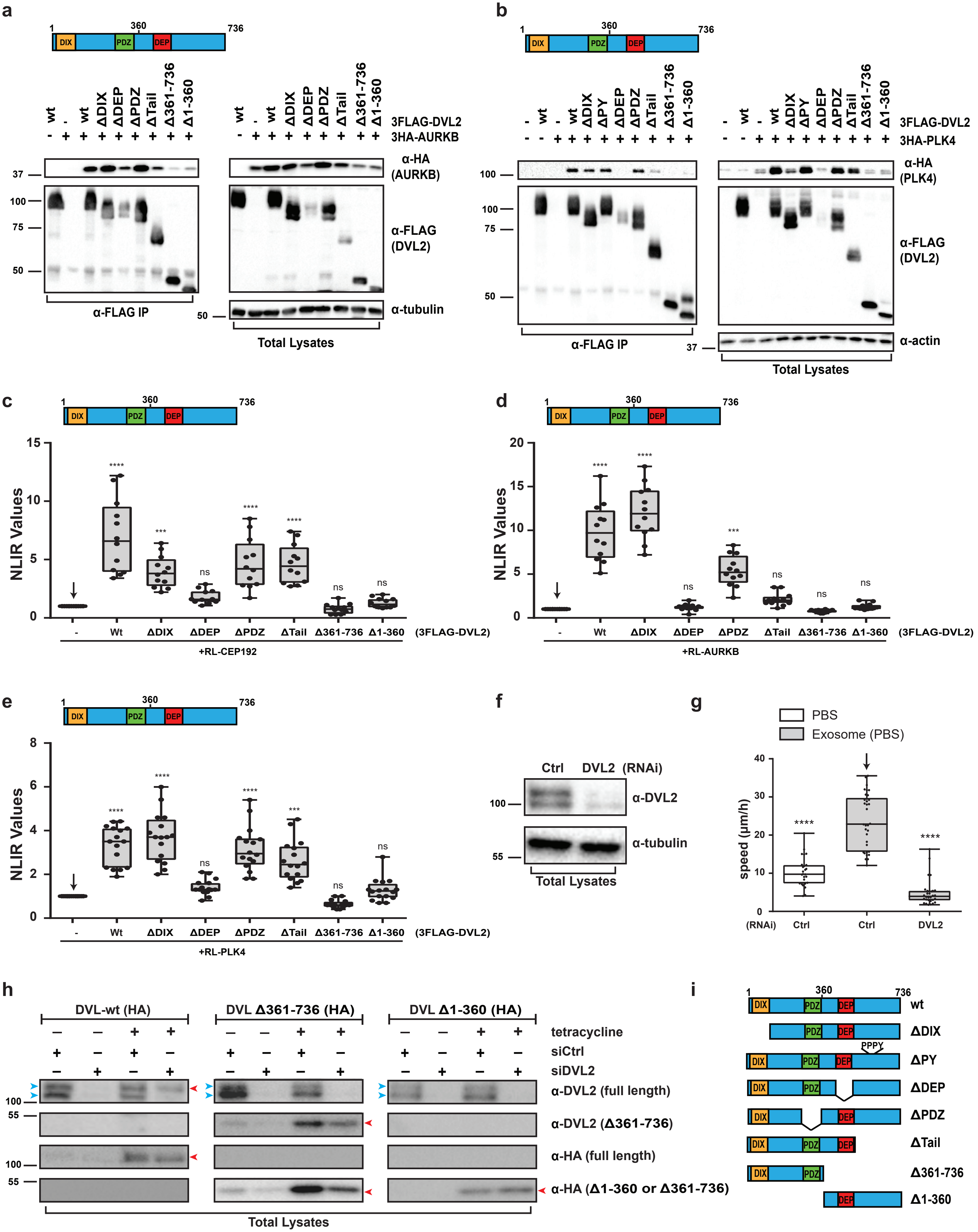

Luo et al., Supplementary Figure 5

Supplementary Figure 5. Domain mapping of DVL2 association with AURKB, PLK4 and CEP192. a, b, HEK293T cells were co-transfected with wild type (wt) 3Flag-DVL2 or its deletion mutants along with 3HA-AURKB (**a**) or 3HA-PLK4 (**b**). Cell lysates were immunoprecipitated with anti-Flag antibody. AURKB or PLK4 were detected by western blotting using anti-HA antibody. (N=3). **c, d, e,** HEK293T cells were co-transfected with wild type (wt) 3Flag-DVL2 or its deletion mutants along with RL-CEP192 (**c**), RL-AURKB (**d**) or RL-PLK4 (**e**). CEP192-, AURKB- or PLK4- Renilla Luciferase activity was measured in manual LUMIER assays (***$p<0.001$, ****$p<0.0001$; N=3). **f,** MDA-MB-231 cells were transfected with control or DLV2 siRNAs for 72h. Cell lysates were blotted for DVL2 or α-tubulin as indicated. (N=3). **g,** MDA-MB-231 cells were transfected with control or DVL2 siRNA for 72h and then stimulated overnight with PBS or purified exosomes resuspended in PBS. Cell motility was measured as described in Methods. (****$p<0.0001$; N=3, at least 30 cells were tracked per condition). **h,** MDA-MB-231 cells stably expressing tetracyclin inducible C-terminally HA-tagged DVL2-wt, DVL2-N term (Δ361-736) or DVL2-C term (Δ1-360) were transfected with control or DVL2 siRNA targeting the 3'UTR region of DVL2. After 72h cells were treated with or without tetracycline. Cell lysates were processed for western blotting with anti-DVL2 and anti-HA antibodies. Blue arrowheads indicate endogenous DVL2 (non-phosphorylated and phosphorylated forms), red arrowheads indicate induced DVL2-HA (or its deletion mutants). (N=3). **i,** Diagram of DVL2 deletion mutants used to determine the domains required for its association with AURKB, PLK4 and CEP192. All data were plotted as box and whiskers. Boxes represent median and 25th to 75th percentiles, whiskers the minimum and maximum values with each individual cell value superimposed. Data were compared with one-way ANOVA Kruskal-Wallis Test and post-tested with Dunn's Multiple Comparison Test. Arrow indicates the control bar used for comparison.

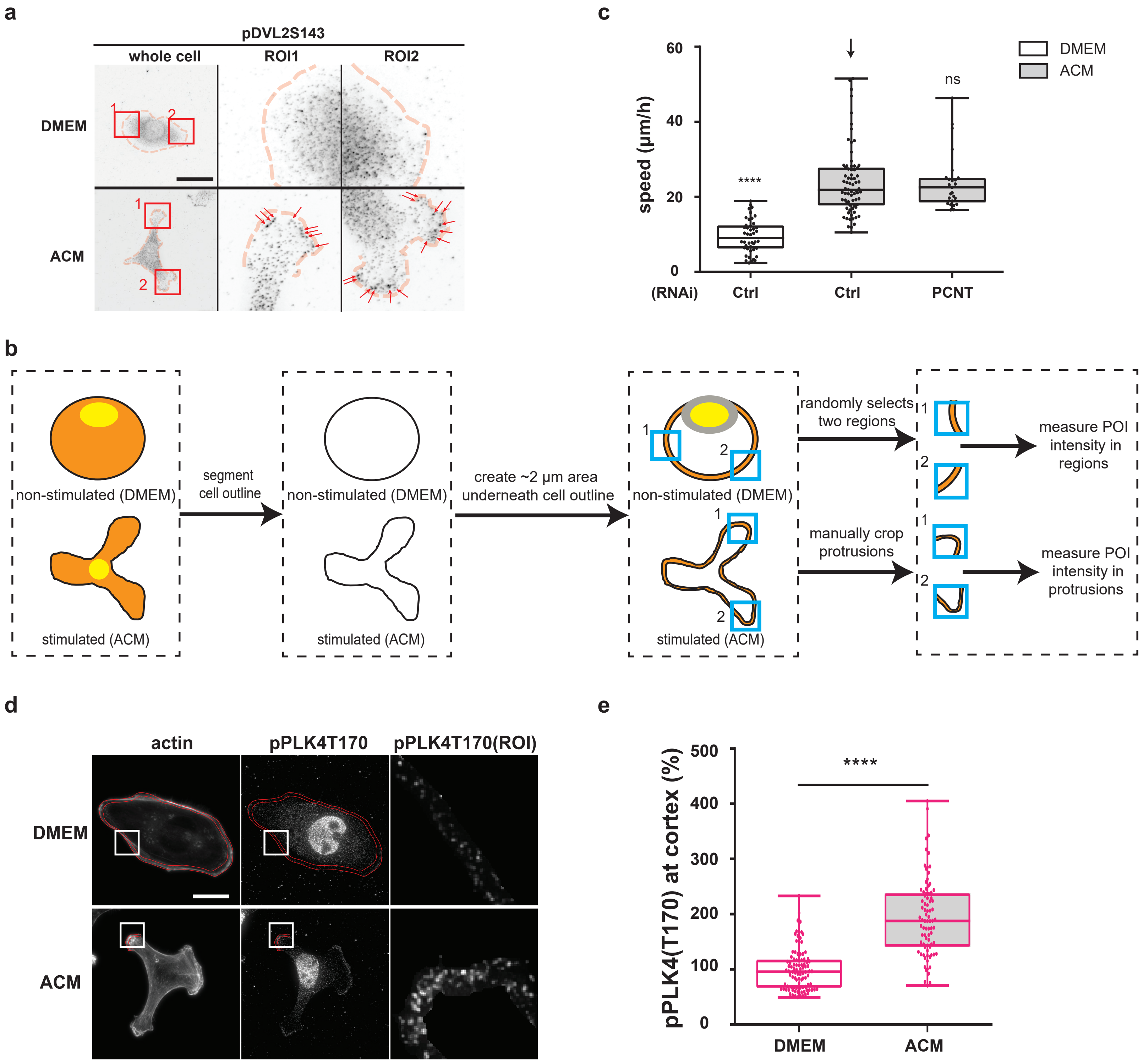

Luo et al., Supplementary Figure 6

Supplementary Figure 6. DVL2 localizes to cell protrusions after ACM stimulation. a, Representative images of MDA-MB-231 cells treated with DMEM or ACM overnight. Immunofluorescence staining for phospho-DVL2 (S143) is shown and cell protrusions were enlarged on the right side panels. Accumulation of phospho-DVL2 (S143) is indicated by red arrows and cell edge is indicated by the orange dash line. Bar=20 µm. (N=3). **b,** Schematics of semi-automated segmentation and quantification of Protein of Interest (POI) average intensity at the cell cortex or cell protrusions. **c**, Cells were transfected with control or PCNT siRNAs for 72h and then treated overnight with DMEM or ACM. Cell motility was measured as described in Methods and plotted as box and whiskers. Boxes represent median and 25th to 75th percentiles, whiskers the minimum and maximum values with each individual cell value superimposed. Data were compared with one-way ANOVA Kruskal-Wallis Test and post-tested with Dunn's Multiple Comparison Test. Arrow indicates the control bar used for comparison. (****p<0. 0001; N=3, at least 30 cells were tracked per condition) **d,** MDA-MB-231 cells were treated with DMEM or ACM overnight and stained for phospho-PLK4 (T170) and actin. Bar=20 µm. **e,** Intensity of phospho-PLK4 (T170) at the cell cortex was measured and plotted as box and whiskers as in (**c**). Data for (**e**) were analyzed by Mann-Whitney U test Two-tailed. (***p<0. 001; N=3, at least 40 cells were measured per condition).

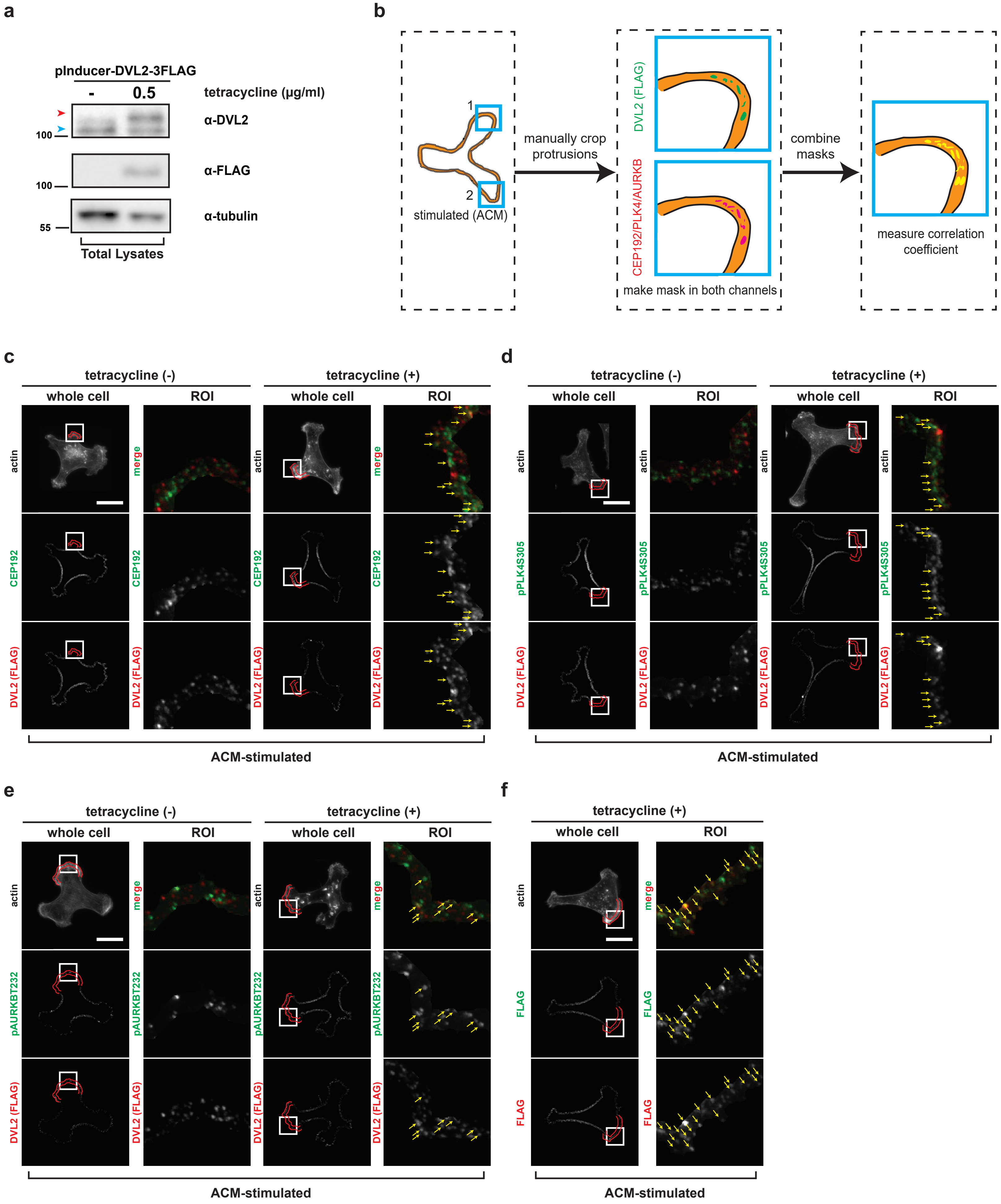

Luo et al., Supplementary Figure 7

g

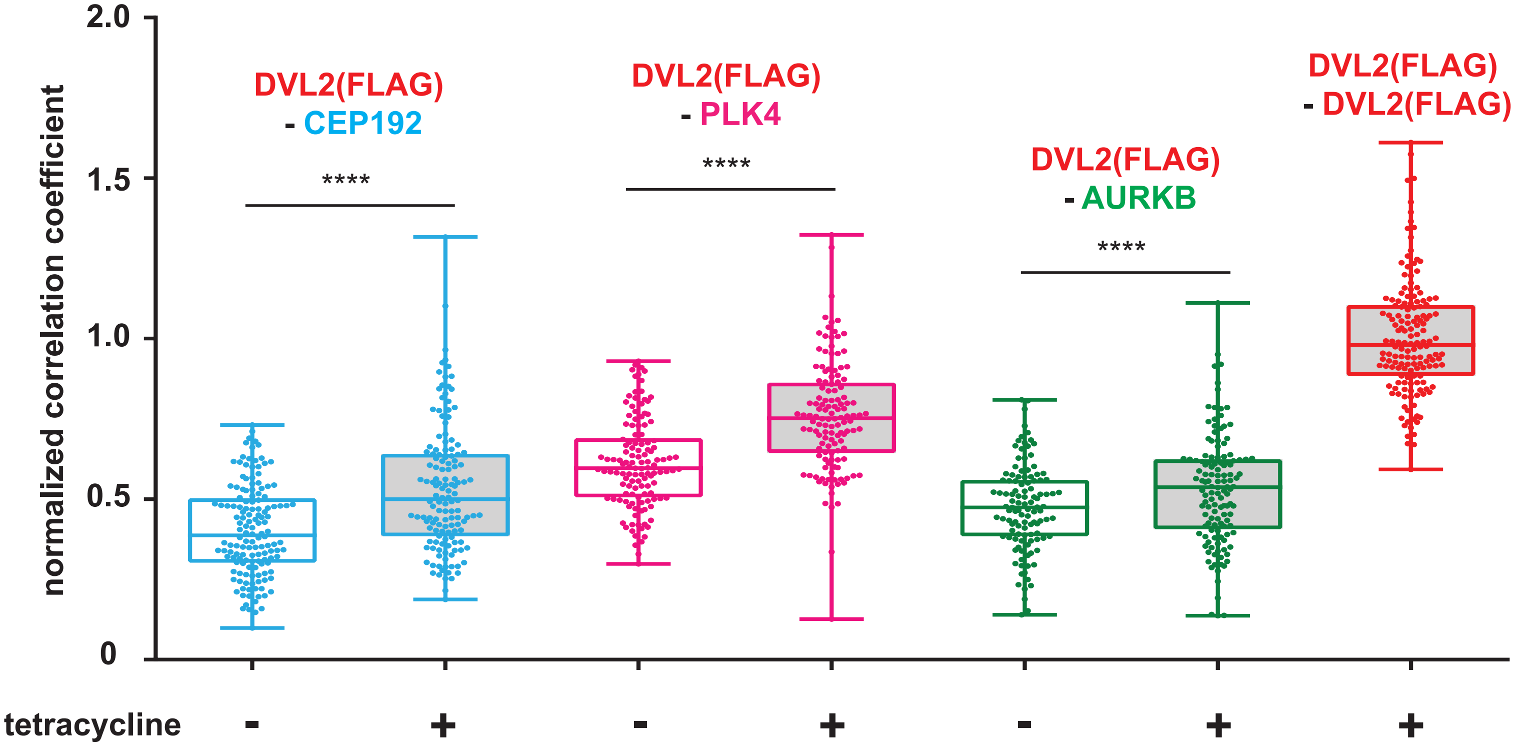

Supplementary Figure 7. DVL2 colocalizes with AURKB, PLK4 and CEP192 at cell protrusions. a, MDA-MB-231 cells stably expressing tetracyclin inducible C-terminally 3Flag-tagged DVL2-wt were treated with 0.5 µg/ml of tetracycline overnight. Cell lysates were processed for western blotting with anti-DVL2, anti-Flag and anti-tubulin antibodies. Blue arrowhead indicates endogenous DVL2, red arrowhead indicates induced DVL2-3Flag. (N=3). **b,** Schematics of semi-automated segmentation and quantification of Proteins of interest (POIs) correlation coefficient at cell protrusions. **c-f,** MDA-MB-231 cells stably expressing tetracyclin inducible 3Flag-tagged DVL2 were incubated with or without 0.5 µg/ml tetracyclin overnight in the presence of ACM. Cells were stained for actin, Flag and (**c**) CEP192, (**d**) phospho-PLK4 (S305), (**e**) phospho-AURKB (T232), or (**f**) Flag (using a different secondary antibody, see Methods). Arrows indicate colocalization areas. Bar=20 µm. **g,** Quantification of normalized correlation coefficient of POIs from **c to f.** Data were plotted as box and whiskers. Boxes represent median and 25th to 75th percentiles, whiskers the minimum and maximum values with each individual cell value superimposed. Data were analyzed by Mann-Whitney U test Two-tailed. (**p<0.01, ****p<0. 0001; N=3, at least 60 cells were measured per condition).

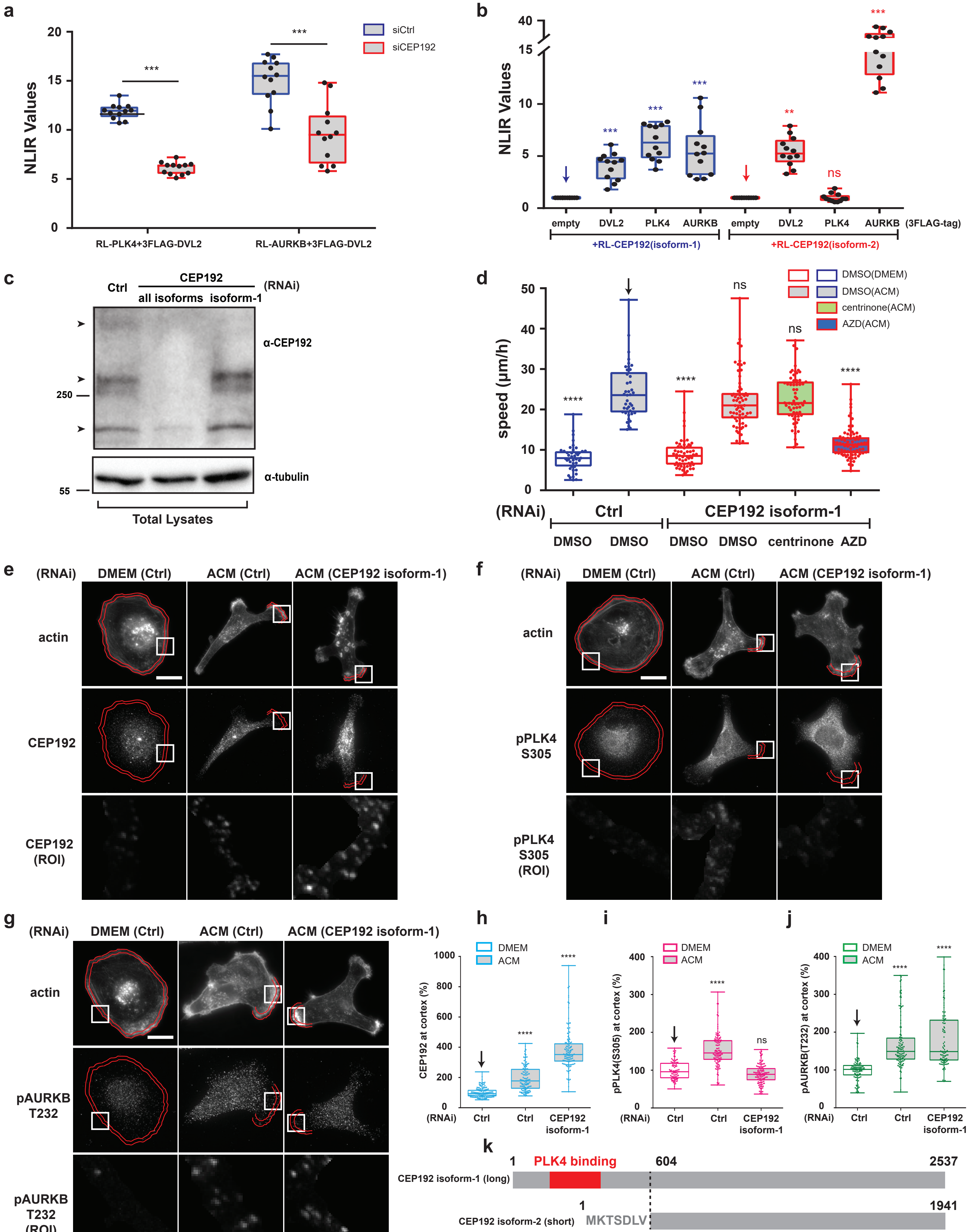

Luo et al., Supplementary Figure 8

Supplementary Figure 8. DVL2 recruits AURKB or PLK4 to cell protrusions via different CEP192 isoforms. a, HEK293T cells were transfected with control or CEP192 siRNAs for 48h, and then co-transfected with 3Flag-DVL2 and RL-PLK4 or RL-AURKB and further incubated for 48h. Cell lysates were immunoprecipitated with anti-Flag antibody in manual LUMIER assays. Data were analyzed by Mann-Whitney U test Two-tailed. (***p<0. 001; N=3). **b,** HEK293T cells were co-transfected with 3Flag empty vector, 3Flag-DVL2, 3Flag-PLK4 or 3Flag AURKB with RL-CEP192 (isoform-1, 1-2537) or RL-CEP192 (isoform-2, 1-1941). Cell lysates were immunoprecipitated with anti-Flag antibody for manual LUMIER assays. (***p<0. 001; N=3). **c,** MDA-MB-231 cells were transfected with the following siRNAs: control, CEP192 (targeting all isoforms) or CEP192 targeting isoform-1 for 72h. Cell lysates were processed for western blotting with anti-CEP192 and anti-tubulin. N=3. **d,** MDA-MB-231 cells were transfected with control or CEP192 isoform-1 siRNAs for 72h and then stimulated overnight with DMEM or ACM with or without centrinone or AZD. Cell motility was measured as described (****p<0. 0001; N=3, at least 40 or cells were tracked per condition). **e-g,** Representative images of MDA-MB-231 cells transfected with control or CEP192 isoform-1 siRNAs as indicated and stimulated overnight with DMEM or ACM. Cells were stained for actin and (**e**) CEP192, (**f**) phospho-PLK4 (S305), or (**g**) phospho-AURKB (T232). Bar=20 µm. **h-j,** Protein signal intensity at cortical region for (**h**) CEP192, (**i**) phospho-PLK4 (S305), or (**j**) phospho-AURKB (T232) was measured from cells in (**e**) to (**g**) and data was analyzed as described in Methods (****p<0. 0001; N=3, at least 60 cells were measured per condition). **k,** Diagram of CEP192 isoform-1 (long) and isoform-2 (short). Isoform-2 (1-1941) is identical to isoform-1 (1-2537) from AA 604-2537, with a different N-terminus (MKTSDLV). Isoform-2 lacks the PLK4 binding site, which corresponds to AA 190-240 in isoform-1 (Red region). All data were plotted as box and whiskers. Boxes represent median and 25th to 75th percentiles, whiskers the minimum and maximum values with each individual cell value superimposed. Data were compared with one-way ANOVA Kruskal-Wallis Test and post-tested with Dunn's Multiple Comparison Test. Arrow indicates the control bar used for comparison.

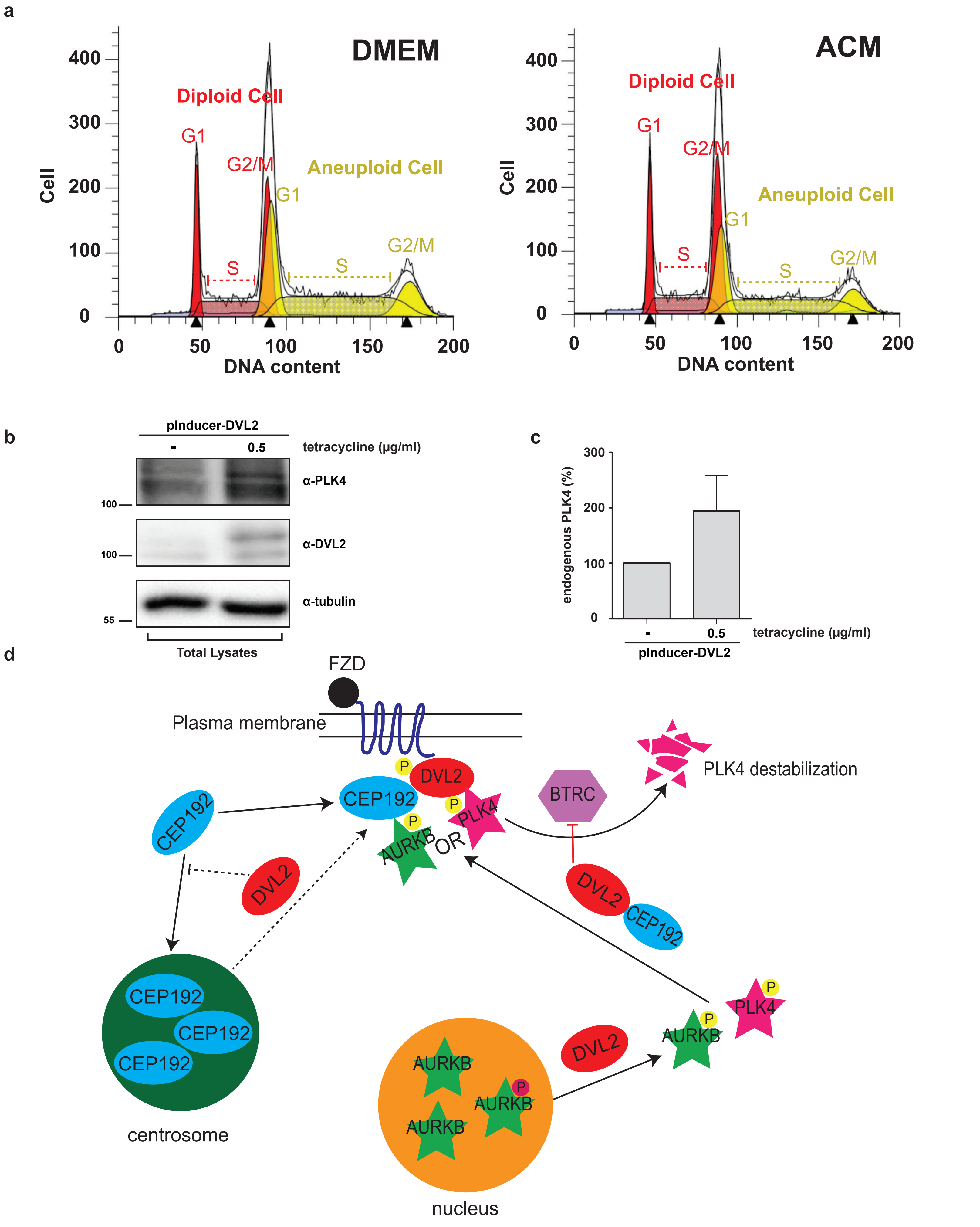

Luo et al., Supplementary Figure 9

Supplementary Figure 9. ACM containing Wnt11-positive exosomes control CEP192, AURKB and PLK4 through DVL2. a, Cell cycle flow cytometry analysis of MDA-MB-231 cells after overnight treatment with DMEM or ACM. (N=2, 2000 cells were measured per condition). **b,** MDA-MB-231 cells stably expressing tetracyclin inducible C-terminally 3Flag-tagged DVL2-wt was incubated with 0.5 μg/ml tetracycline overnight. Cell lysates were processed for western blotting with anti-PLK4, anti-DVL2 and anti-tubulin. **c,** Quantification of endogenous PLK4 levels with or without induced DVL2 expression from (**b**). Data were plotted as mean ± s.e.m, ($p=0.125$, $N=3$) Data were analyzed by Mann-Whitney U test One-tailed. **d,** Model for DVL2 regulating recruitment of CEP192, AURKB and PLK4 to cell cortex in response to ACM stimulation.

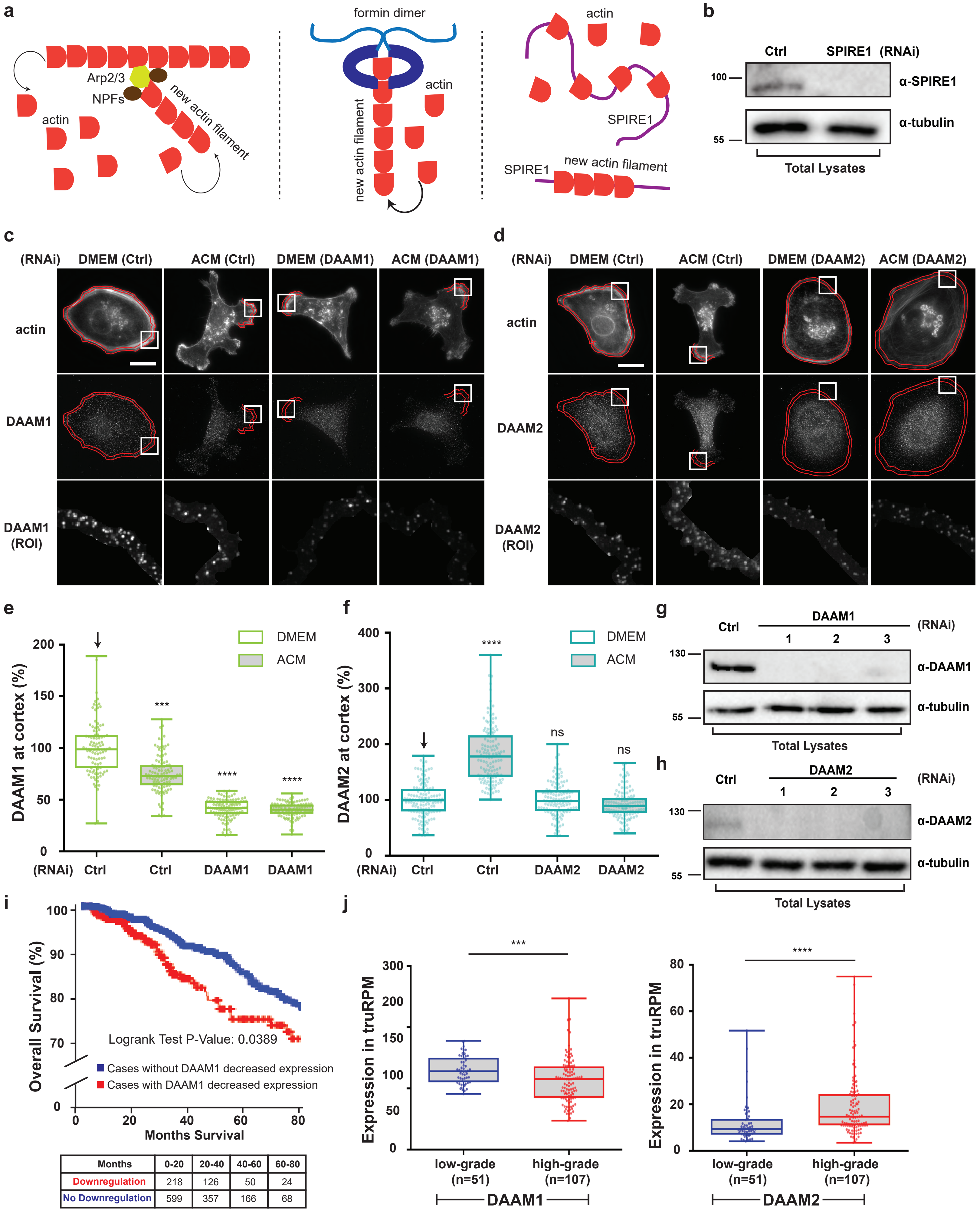

Months	0-20	20-40	40-60	60-80
Downregulation	218	126	50	24
No Downregulation	599	357	166	68

(Total Patient Number Involved In Program)

Luo et al., Supplementary Figure 10

Supplementary Figure 10. DAAM1 decreased expression and DAAM2 increased expression correlates with poor cancer prognosis and survival. a, Schematic summary of the three major actin nucleation pathways: ARP2/3, formins and SPIRE1. **b,** MDA-MB-231 cells were transfected with control or SPIRE1 siRNA for 72h. Cell lysates were processed for western blotting with SPIRE1 or α-tubulin antibodies as indicated. (N=3). **c, d,** Representative images of MDA-MB-231 cells transfected with control, DAAM1 or DAAM2 siRNAs for 72h, and subsequently stimulated overnight with DMEM or ACM. Cells were stained for actin and (**c**) DAAM1, or (**d**) DAAM2. Bar=20 µm. **e, f,** Intensity of (**e**) DAAM1 and (**f**) DAAM2 at the cortical region, which was measured and plotted as box-and-whiskers. Boxes represent median and 25th to 75th percentiles, whiskers the minimum and maximum values with each individual cell value superimposed. Data were compared with one-way ANOVA Kruskal-Wallis Test and post-tested with Dunn's Multiple Comparison Test, Arrow indicates the control bar used for comparison. (***$p<0.001$, **** $p<0.0001$; N=3, at least 60 cells were measured per condition). **g, h,** MDA-MB-231 cells were transfected with control siRNA, (**g**) DAAM1 or (**h**) DAAM2 individual siRNA oligos for 72h used in **Fig. 9c**. Cell lysates were processed for western blotting with DAAM1, DAAM2 or α-tubulin antibodies as indicated. **i,** Survival data of breast invasive carcinoma patients with or without decreased expression (shallow depletion) in DAAM1 (top panel). Total number of patients involved in the program at each time period is indicated in the bottom table. Graph is based on data from TCGA Research Network[68,69] (TCGA Provisional, http://www.cbioportal.org). **j,** RNA expression levels for DAAM1 and DAAM2 in low grade and high-grade bladder cancer patient samples analyzed by RNA-seq as described in Methods and plotted as box-and-whiskers as in **e** and **f**. Number of patients from each group is indicated in X axis. Data were analyzed by Mann-Whitney U test Two-tailed (*** $p<0.001$, **** $p<0.0001$).

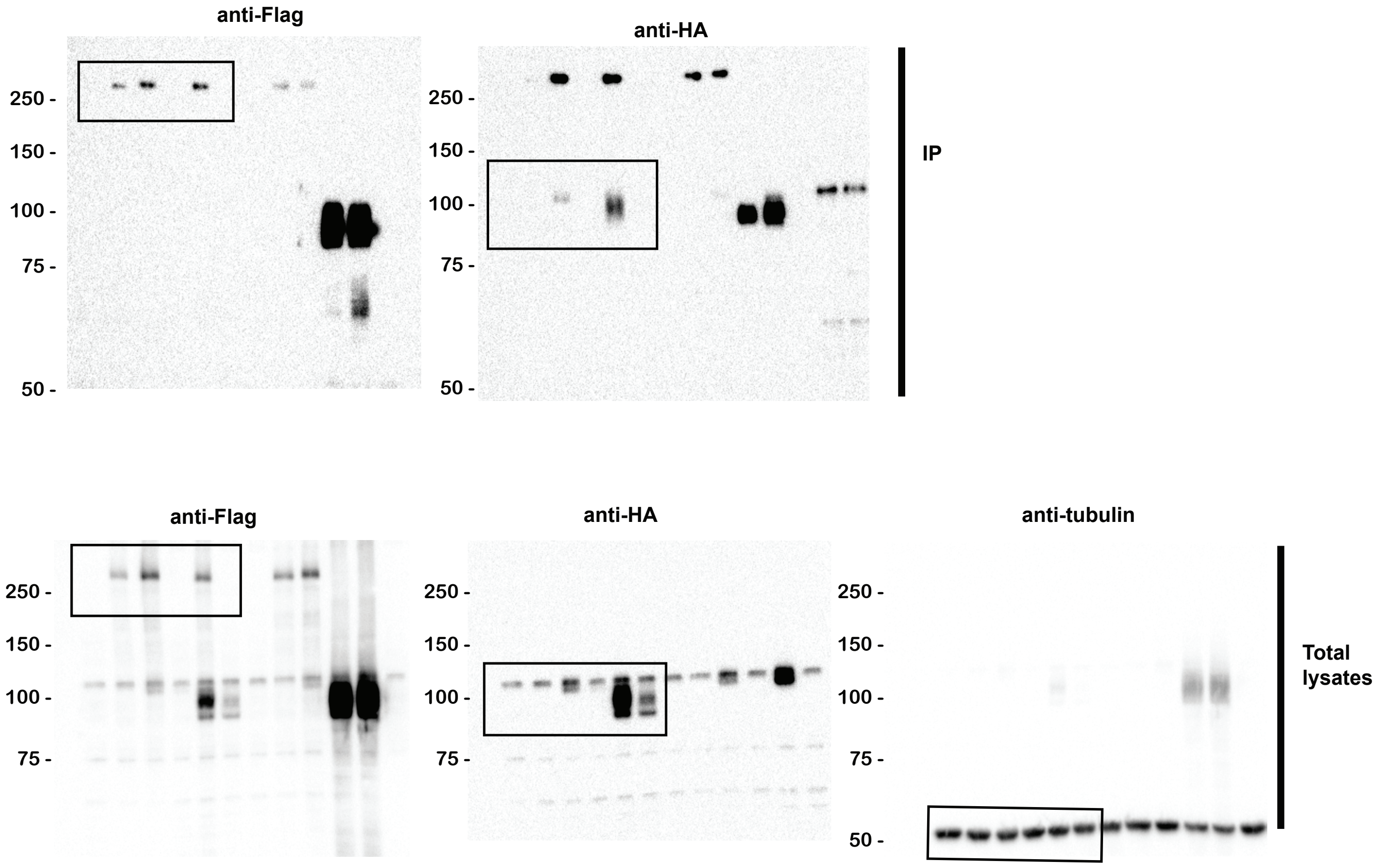

The boxes indicate the blots presented in Fig. 4b.

Luo et al., Supplementary Figure 11

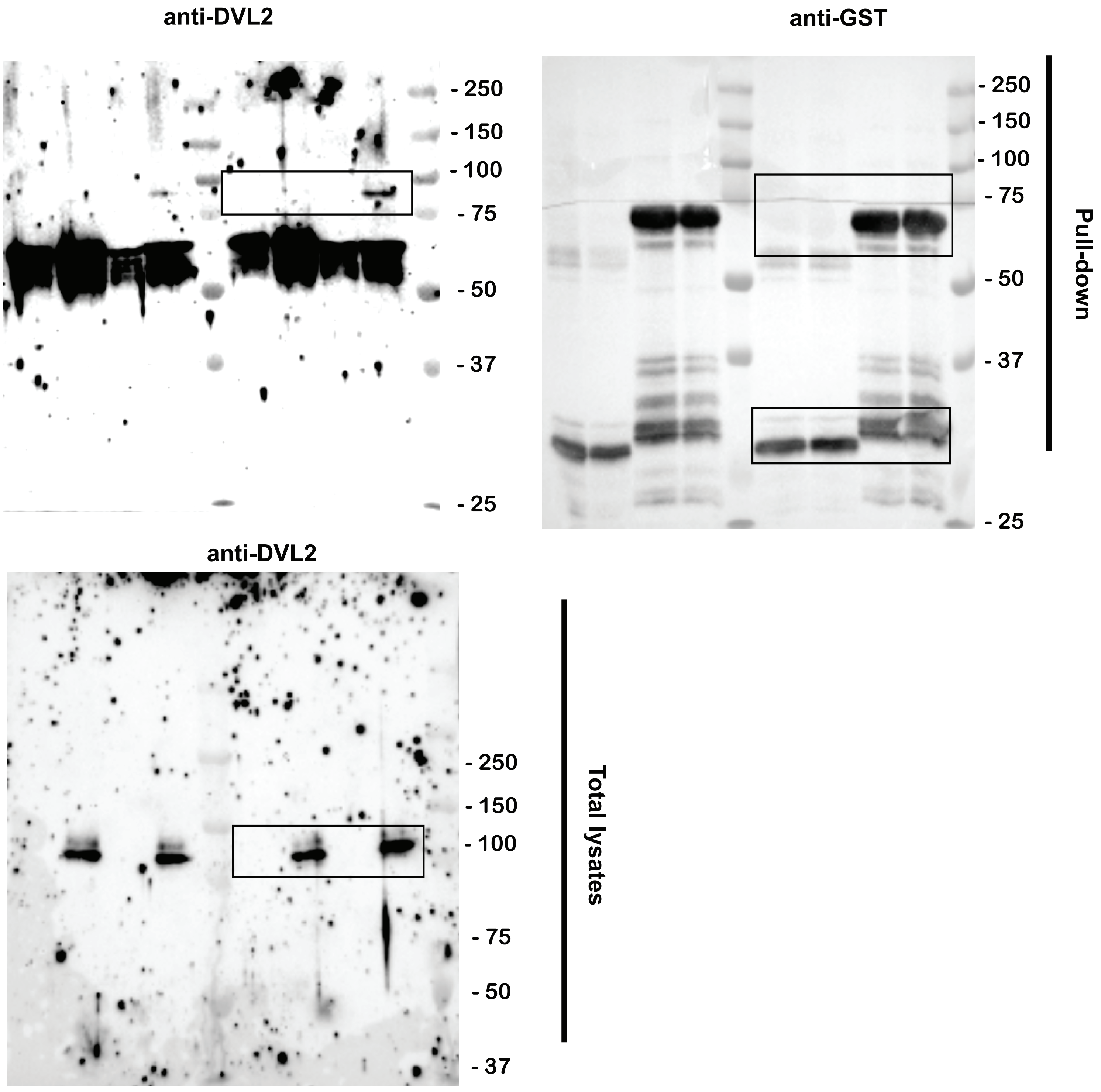

The boxes indicate the blots presented in Fig. 4c.

Luo et al., Supplementary Figure 11

anti-DVL2

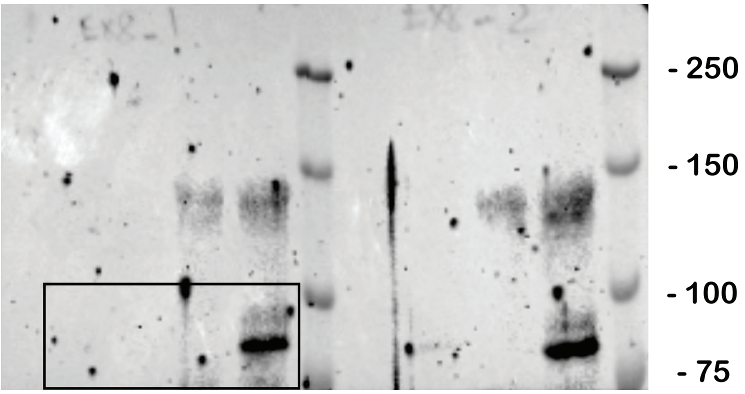

anti-GST

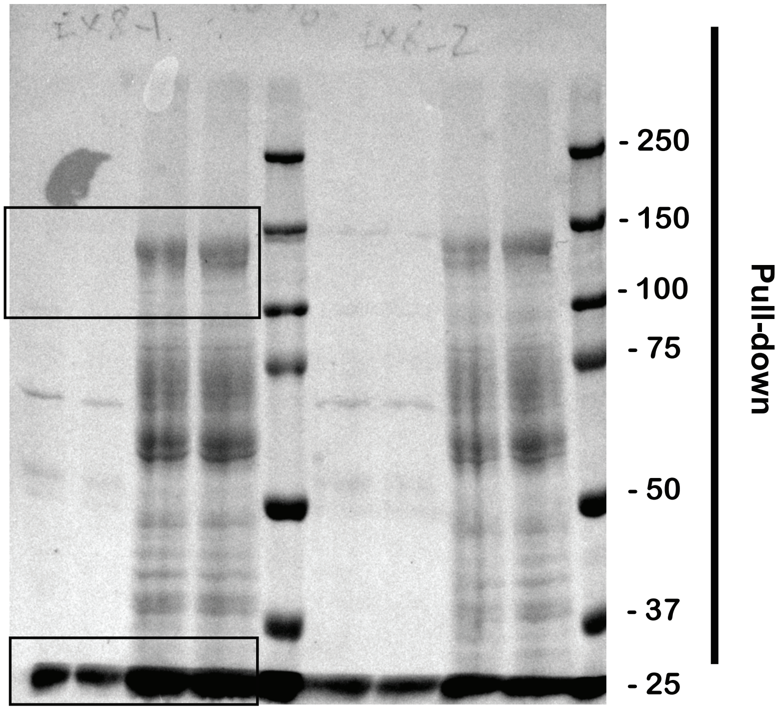

anti-DVL2

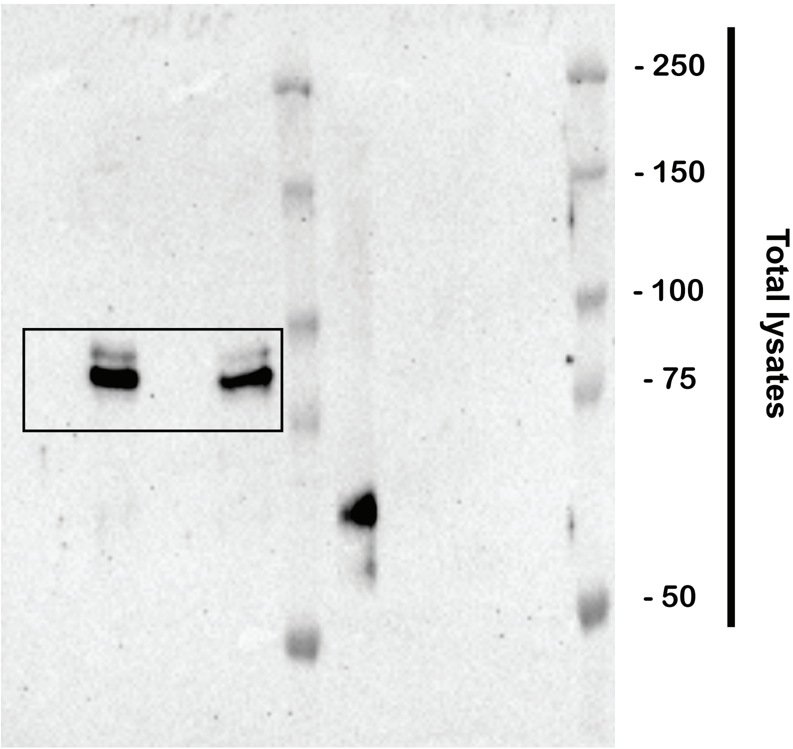

The boxes indicate the blots presented in Fig. 4d.

Luo et al., Supplementary Figure 11

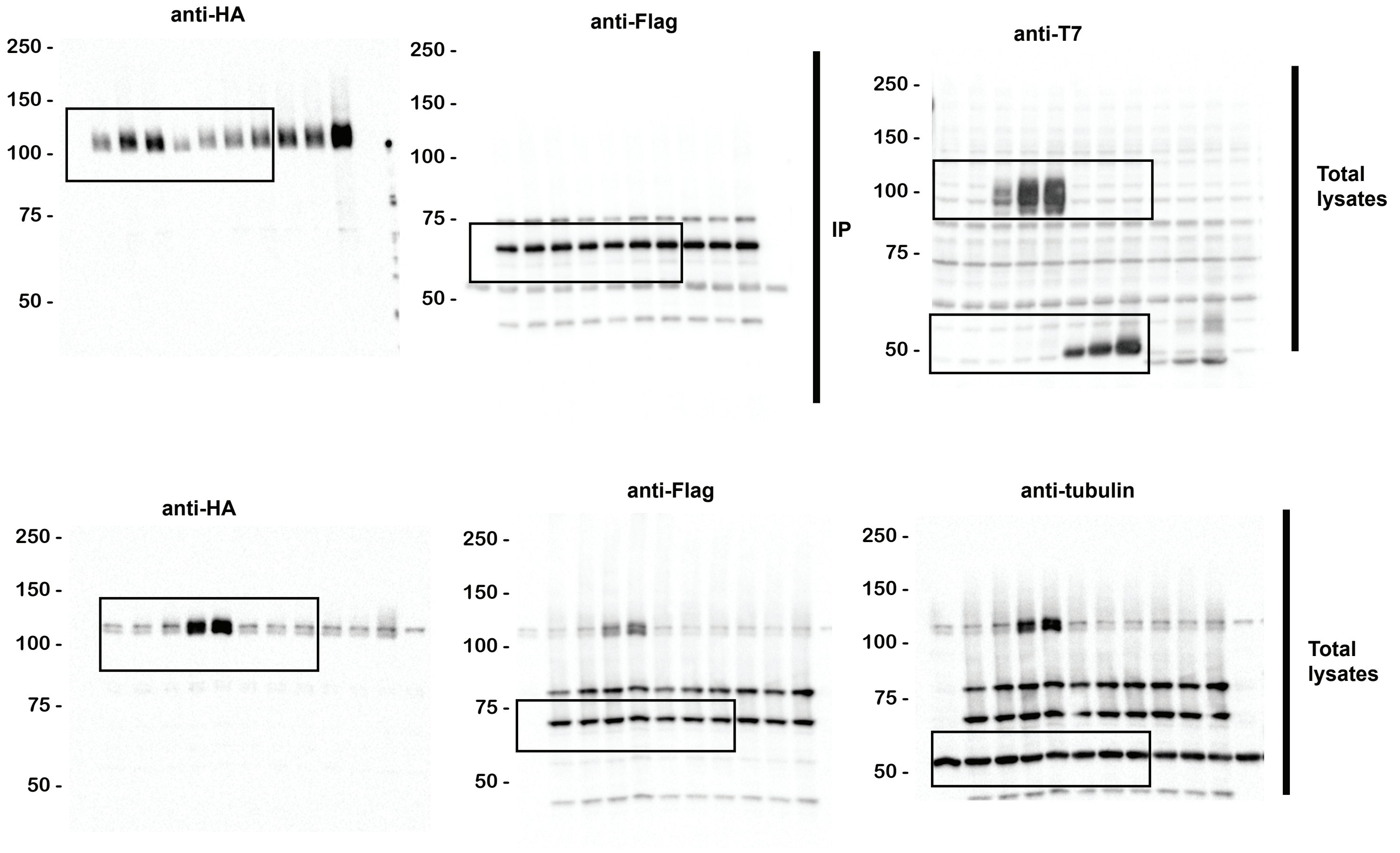

The boxes indicate the blots presented in Fig. 8g.

Supplementary Figure 11. Uncropped blots from Fig.4 and Fig.8.

Supplementary Table 1. NLIR values of LUMIER screens

		CEP192		PLK4		AURKB		AURKC	
Flag-tagged Preys		NLIR_1	NLIR_2	NLIR_1	NLIR_2	NLIR_1	NLIR_2	NLIR_1	NLIR_2
**1**	CEP192	16.2	13.8	18.0	17.3	2.1	3.0	3.5	3.9
**2**	PLK4	15.1	6.7	45.4	38.3	2.9	3.6	2.6	4.0
**3**	AURKC	5.4	4.8	5.0	4.7	4.3	4.6	5.5	7.3
**4**	AURKB	8.1	5.5	5.0	5.0	25.4	22.7	4.9	6.5
**5**	AURKA	25.1	18.8	3.3	2.7	2.8	2.6	1.6	1.6
**6**	Axin2	0.7	0.9	2.5	3.0	2.3	3.2	4.1	3.9
**7**	AXIN1	0.7	0.9	9.5	9.6	4.4	4.1	4.3	5.0
**8**	CUL1	1.0	1.2	7.0	5.3	1.3	1.7	1.3	1.8
**9**	DVL2	6.7	6.3	9.0	9.5	26.9	29.1	24.7	30.7
**10**	Lef1	1.0	1.0	1.3	1.3	1.4	1.5	1.2	1.6
**11**	PIAS1	1.7	1.4	3.3	2.8	1.9	2.0	2.0	2.3
**12**	PIAS2	0.9	0.8	1.1	1.1	1.2	1.2	1.2	1.1
**13**	Pias4	0.5	0.5	0.8	0.9	1.8	1.9	2.6	2.4
**14**	SUMO3	1.2	1.4	1.7	1.5	1.0	2.1	1.6	1.3
**15**	TCF7 isoform b	1.8	2.3	1.9	2.1	1.5	2.4	1.9	1.9
**16**	TCF7 isoform e	1.3	1.7	2.8	1.2	1.5	1.3	1.2	1.5
**17**	AKT1	0.7	0.9	1.6	1.9	4.0	5.3	6.5	6.4
**18**	PPP2R5A	0.8	0.7	1.2	1.9	1.7	1.7	1.5	2.1
**19**	PPP2R5D	1.2	1.4	2.6	2.8	1.8	2.1	2.1	2.3
**20**	ARRB1	0.4	0.5	0.7	1.0	1.5	1.4	1.8	2.2
**21**	PRKCI	0.8	0.7	0.8	0.9	3.7	3.6	3.7	3.7
**22**	CSNK1A1	7.6	9.2	5.8	6.3	3.4	4.1	7.2	7.4
**23**	Csnk2b	1.5	2.0	2.2	2.7	2.1	2.3	2.9	2.9
**24**	SORBS3	0.6	0.5	0.5	0.6	1.8	1.7	1.4	1.6
**25**	EPS8	0.8	1.0	1.2	1.3	1.4	1.5	1.0	1.2
**26**	FHL2	2.2	2.2	3.2	4.0	3.6	2.7	1.8	2.5
**27**	FRAT1	2.8	2.8	2.1	1.9	3.6	3.0	3.3	4.6
**28**	CTNNBIP1	1.3	0.7	4.7	4.2	3.2	2.7	1.7	1.8
**29**	NKD2-variant1(v1)	0.6	0.5	1.3	1.2	4.6	3.4	5.1	5.3
**30**	TP53	0.1	0.2	0.3	0.3	1.4	0.4	0.8	1.6
**31**	PTEN	0.8	1.0	1.0	1.6	1.8	2.2	1.9	2.3
**32**	SKP1	1.1	3.1	2.9	9.4	1.6	15.2	1.2	1.6
**33**	GSK3B	3.0	4.5	4.6	5.6	4.5	5.3	5.9	6.6
**34**	PPP2R5C	1.1	2.1	1.2	3.8	1.5	2.6	1.3	1.2
**35**	DAB2	1.0	1.7	1.3	1.6	1.7	1.5	1.4	1.5
**36**	Pik3r2	1.0	1.8	2.8	4.8	3.7	4.6	4.8	4.2
**37**	Tcf3	0.4	0.3	0.4	0.6	1.2	1.3	2.7	1.8
**38**	PPP2R5B	0.4	0.3	0.6	0.6	1.6	1.7	1.4	1.8
**39**	ANKRD6	1.4	1.5	3.7	11.1	2.2	3.6	2.1	4.0
**40**	DVL1	0.2	0.2	1.0	1.1	1.9	3.0	1.0	1.4
**41**	TBL1X	0.8	1.2	1.4	3.8	2.8	2.3	7.5	2.8
**42**	NKD1	2.1	3.0	8.9	8.2	7.0	5.6	7.2	5.3
**43**	SIAH1	1.2	1.3	1.2	1.4	1.8	1.7	1.3	1.3
**44**	CSNK1E	3.6	3.3	5.0	4.8	13.1	10.9	16.3	16.8
**45**	IKBKB	6.5	7.7	8.6	9.2	5.4	5.2	8.2	9.7
**46**	CACYBP	0.5	0.5	0.7	3.0	1.6	1.9	1.8	2.2
**47**	STK11	3.4	4.5	5.0	3.8	11.9	10.1	21.9	23.9
**48**	Senp2	4.0	4.7	3.8	2.5	8.3	4.4	5.3	9.4
**49**	BTRC	4.8	4.1	12.3	9.8	5.6	4.7	5.1	6.5
**50**	Dlg5	3.4	4.3	5.6	5.3	4.9	3.6	4.5	8.5
**51**	DVL3	1.9	1.6	11.5	12.3	14.2	11.9	8.3	8.5
**52**	Fyn	0.5	0.4	0.4	0.6	1.8	1.5	1.3	1.5
**53**	Tp53bp1	0.7	0.7	0.9	1.2	2.7	2.8	4.5	6.2
**54**	PIK3R1	2.1	2.8	2.9	3.3	3.8	3.3	3.8	4.6
**55**	TAX1BP3	0.6	0.5	1.1	0.9	1.6	1.9	0.9	1.3
**56**	Ube2r2	0.6	0.7	0.9	1.0	4.4	3.9	4.5	5.1
**57**	CTNNA1	0.5	0.5	0.5	0.7	2.3	1.9	0.8	1.2
**58**	Frat2	0.8	0.9	1.1	1.2	1.9	1.7	0.9	1.5
**59**	PPP2CB-variant1(v1)	2.8	2.8	8.3	7.9	7.8	6.1	6.9	8.9
**60**	PPP2CB	0.8	0.9	1.3	1.5	4.0	2.9	4.5	4.4
**61**	SIAH2	0.6	0.5	0.6	0.6	1.3	1.5	1.3	1.4
**62**	ELAVL1	0.9	0.8	0.8	0.9	2.9	2.8	3.2	3.4
**63**	Kap	1.7	2.1	3.3	2.6	3.3	2.7	2.6	3.1
**64**	KIF1B	2.4	2.8	7.8	6.7	10.3	10.4	17.1	13.0
**65**	KIF2A	1.1	1.6	1.4	1.2	2.2	2.0	1.1	1.5
**66**	KLC1	2.0	2.6	1.9	2.3	2.8	1.8	1.5	1.8
**67**	GSK3A	3.5	4.0	5.9	6.3	4.3	4.2	6.2	5.4
**68**	WISP2	3.0	3.4	5.5	6.8	3.2	3.0	3.3	3.2
**69**	PTK2	1.1	1.2	2.0	1.6	2.3	2.5	3.0	3.8
**70**	CDK2	0.9	0.8	1.0	1.0	2.4	2.0	1.8	2.2
**71**	CCNE2	1.5	1.9	2.5	2.0	2.1	2.5	1.8	2.7
**72**	Ikbke	2.0	1.9	3.6	3.3	15.3	14.6	19.9	24.9
**73**	VCL	0.7	0.8	1.0	0.7	1.6	1.6	0.9	1.1
**74**	Fzd7	1.0	1.1	1.4	1.4	2.7	2.4	1.9	2.1
**75**	PTPN1	0.8	1.0	0.9	0.9	3.2	2.8	3.5	5.3
**76**	PTPN6	0.6	0.6	2.1	0.6	1.5	1.4	1.5	1.4
**77**	INTU	2.5	3.1	4.9	5.9	6.8	7.5	18.9	15.8
**78**	MPP2	3.0	2.4	1.9	1.9	3.7	3.6	2.9	3.1
**79**	GOPC	1.9	1.6	2.1	1.9	20.2	2.9	2.6	3.3
**80**	GPSM2	0.5	0.5	1.2	2.6	2.1	2.4	2.8	5.4
**81**	ACP1	1.9	3.9	2.7	2.5	3.6	4.7	3.7	4.5
**82**	Nkd2	2.4	3.0	2.9	2.9	6.3	6.6	10.5	10.9
**83**	SNRPN	0.2	0.3	0.5	0.5	1.5	1.5	1.0	1.3
**84**	Prkaca	3.1	3.0	6.4	5.1	5.4	5.3	6.7	6.6

Supplementary Table 1. NLIR values from two independent LUMIER screens using Renilla luciferase-tagged CEP192 (isoform-1), PLK4, AURKB and AURKC and 84 3Flag-tagged preys. Pearson Correlation Coefficient between screen replicates r=0.93. Positive control bait interactors are underlined.

Supplementary Table 2. RNAi sequences used in this study.

Gene	Species	Name	Target Sequence
**Dharmacon On-Target plus SMART pool siRNA**			
SPIRE1	human	siSPIRE1#1	5'-AAAGAUACACCUUGCGAAA
SPIRE1	human	siSPIRE1#2	5'-CCAGAGGAGAUUAGACGUA
SPIRE1	human	siSPIRE1#3	5'-CCUCAAAACUGCUCCGGUA
SPIRE1	human	siSPIRE1#4	5'-AAAUUAAGAGUGCGAAAGA
CEP192	human	siCEP192#1	5'-UGUGAAGAAAUACGAGAUA
CEP192	human	siCEP192#2	5'-GCUCAGCGGUAUUUGGGAA
CEP192	human	siCEP192#3	5'-GUCUAGAACUCGAGAAUCA
CEP192	human	siCEP192#4	5'-GGUUGAAGCAGUAGAGAGU
NEDD1	human	siNEDD1#1	5'-UGGGCAGUGUUUCGGAUAA
NEDD1	human	siNEDD1#2	5'-CAUUGGAAAUAACCGGCAA
NEDD1	human	siNEDD1#3	5'-AAUUGAAAGACUACGAGAA
NEDD1	human	siNEDD1#4	5'-CCAUAGGGACAUUGUGAAU
PRICKLE1	human	siPRICKLE1#1	5'-GGACAGAGGUAUAUCAUGA
PRICKLE1	human	siPRICKLE1#2	5'-GAAGAUAAAUGGAGGUGAA
PRICKLE1	human	siPRICKLE1#3	5'-GGGCUGAUCAUGAAGAUUA
PRICKLE1	human	siPRICKLE1#4	5'-GAGAGAAGCAUCGGAUUAA
**Dharmacon siGENOME MARTpool siRNA**			
PLK4	human	siPLK4#1	5'-GAAAUGAACAGGUAUCUAA
PLK4	human	siPLK4#2	5'-GAAACAUCCUUCUAUCUUG
PLK4	human	siPLK4#3	5'-GUGGAAGACUCAAUUGAUA
PLK4	human	siPLK4#4	5'-GGACCUUAUUCACCAGUUA
