# Supplementary material for: Atypical function of a centrosomal module in WNT signalling drives contextual cancer cell motility
Source: Nat Commun. 2019 May 29;10:2356. doi: 10.1038/s41467-019-10241-w (PMC6541620; doi:10.1038/s41467-019-10241-w)
Supplement: Supplementary file 6 — Reporting Summary [file 41467_2019_10241_MOESM6_ESM.pdf]

nature research

Corresponding author(s): Dr. Laurence Pelletier

Last updated by author(s): Apr 19, 2019

Reporting Summary

Nature Research wishes to improve the reproducibility of the work that we publish. This form provides structure for consistency and transparency in reporting. For further information on Nature Research policies, see Authors & Referees and the Editorial Policy Checklist.

Statistics

For all statistical analyses, confirm that the following items are present in the figure legend, table legend, main text, or Methods section.

n/a	Confirmed	
☐	☒	The exact sample size (*n*) for each experimental group/condition, given as a discrete number and unit of measurement
☐	☒	A statement on whether measurements were taken from distinct samples or whether the same sample was measured repeatedly
☐	☒	The statistical test(s) used AND whether they are one- or two-sided<br>*Only common tests should be described solely by name; describe more complex techniques in the Methods section.*
☐	☒	A description of all covariates tested
☐	☒	A description of any assumptions or corrections, such as tests of normality and adjustment for multiple comparisons
☐	☒	A full description of the statistical parameters including central tendency (e.g. means) or other basic estimates (e.g. regression coefficient) AND variation (e.g. standard deviation) or associated estimates of uncertainty (e.g. confidence intervals)
☐	☒	For null hypothesis testing, the test statistic (e.g. *F*, *t*, *r*) with confidence intervals, effect sizes, degrees of freedom and *P* value noted<br>*Give P values as exact values whenever suitable.*
☒	☐	For Bayesian analysis, information on the choice of priors and Markov chain Monte Carlo settings
☒	☐	For hierarchical and complex designs, identification of the appropriate level for tests and full reporting of outcomes
☐	☒	Estimates of effect sizes (e.g. Cohen's *d*, Pearson's *r*), indicating how they were calculated

Our web collection on statistics for biologists contains articles on many of the points above.

Software and code

Policy information about availability of computer code

Data collection	No custom software was used to collect the data.
Data analysis	We can provide the MATLAB code used in this study upon request.

For manuscripts utilizing custom algorithms or software that are central to the research but not yet described in published literature, software must be made available to editors/reviewers. We strongly encourage code deposition in a community repository (e.g. GitHub). See the Nature Research guidelines for submitting code & software for further information.

Data

Policy information about availability of data

All manuscripts must include a data availability statement. This statement should provide the following information, where applicable:

- Accession codes, unique identifiers, or web links for publicly available datasets
- A list of figures that have associated raw data
- A description of any restrictions on data availability

The authors declare that all data supporting the findings of this study can be found within the paper and its Supplementary Information files or from the corresponding author upon request. The RNA-seq data has been uploaded into the GEO database with provisional accession number GSE129871. The corresponding final accession number will be included in the manuscript before publication. The MATLAB code used in this study is also available from the corresponding author if requested.

Field-specific reporting

Please select the one below that is the best fit for your research. If you are not sure, read the appropriate sections before making your selection.

☒ Life sciences ☐ Behavioural & social sciences ☐ Ecological, evolutionary & environmental sciences

For a reference copy of the document with all sections, see nature.com/documents/nr-reporting-summary-flat.pdf

Life sciences study design

All studies must disclose on these points even when the disclosure is negative.

Sample size	Sample size was described in each figure legend. We randomly selected around 30-60 cell in each experiment with 3-4 independent repeats to conduct appropriate statistical analysis.
Data exclusions	No data were excluded.
Replication	All attempts at replication were successful.
Randomization	Samples were randomly selected.
Blinding	Data collection and analysis was blinded between at least two investigators.

Reporting for specific materials, systems and methods

We require information from authors about some types of materials, experimental systems and methods used in many studies. Here, indicate whether each material, system or method listed is relevant to your study. If you are not sure if a list item applies to your research, read the appropriate section before selecting a response.

Materials & experimental systems

n/a	Involved in the study	
☐	☒	Antibodies
☐	☒	Eukaryotic cell lines
☒	☐	Palaeontology
☒	☐	Animals and other organisms
☒	☐	Human research participants
☐	☒	Clinical data

Methods

n/a	Involved in the study	
☒	☐	ChIP-seq
☐	☒	Flow cytometry
☒	☐	MRI-based neuroimaging

Antibodies

Antibodies used	Phospho-AURKB (T232), BioLegend, 636101;<br>Phospho-DVL2 (S143), Abcam, ab124933;<br>PCNT, Abcam, ab4448;<br>AURKA, Abcam, ab12875;<br>AURKB, BD Biosciences, 611082;<br>PLK4, Millipore, MABC544;<br>Alpha-tubulin, Sigma, T9026;<br>Gamma-tubulin, Sigma, T6557;<br>Beta-actin, Sigma, A5316;<br>GST, Sigma, G7781;<br>Flag, Sigma, F1804;<br>HA-HRP, Sigma, 11867423001;<br>DAAM1, Santa Cruz, sc-100942;<br>DVL2, Cell Signaling Technology, 3324;<br>DAAM2, Novus Biologicals, NBP2-47496;<br>SPIRE1, Novus Biologicals, H00056907-M01;<br>Alexa FluroTM 488 Phalloidin, ThermoFisher, A12379.
Validation	AURKA, AURKB, PLK4, DVL2, DAAM1, DAAM2 and SPIRE1 antibodies were validated by transfecting siRNA targeting the specific proteins and observing the depletion of the protein by western blotting. Phospho-PLK4 (S305) , phospho-PLK4 (T170), phospho-AURKB (T232), phospho-DVL2 (S143), PCNT, AURKB, alpha-tubulin, gamma-tubulin, DAAM1, DAAM2 and Alexa FluroTM 488 Phalloidin were confirmed by IF.

Eukaryotic cell lines

Policy information about cell lines

Cell line source(s)	MDA-MB-231 cells were a gift from Dr. Robert S. Kerbel (Sunnybrook Health Sciences Centre, Toronto, Canada;<br>L cells (CRL-2648™), HEK293T (CRL-3216™) and MDA-MB-468 cells (HTB-132™) were purchased from ATCC ;<br>Mouse breast cancer cell lines EpRas were a gift from Dr. Martin Oft (ARMO BioSciences, Redwood City, California, United States);<br>Human bladder cancer cell lines T24 (HTB-4™) and TCCSUP (HTB-5™) were purchased from ATCC.
Authentication	All cell lines used in this study were authenticated by STR profiling.
Mycoplasma contamination	During the course of this study all cell lines were routinely tested and all of them were free of mycoplasma contamination.
Commonly misidentified lines<br>(See ICLAC register)	None of the cell lines used in this study are in the ICLAC registry

Clinical data

Policy information about clinical studies

All manuscripts should comply with the ICMJE guidelines for publication of clinical research and a completed CONSORT checklist must be included with all submissions.

Clinical trial registration	A research study using archival bladder cancer samples was conducted with ethics board approval from two institutions: University Health Network 11-0134-T and Mount Sinai Hospital 11-0015-E
Study protocol	RNA used for RNAseq analysis was extracted from archival bladder cancer FFPE tumor samples. The protocol was reported in Liu Y. et al. Eur. Urol. 66:982-986 (2014). cDNA libraries were prepared form total RNA from tumor samples and subjected to Next Generation Sequencing following Illumina guidelines and using a HiSeq2000 platform.
Data collection	FFPE bladder cancer samples had been archived and stored at the University Health Network (Toronto, Canada). The RNA extraction and Next Generation Sequencing was conducted at the Lunenfeld Research Institute of Mount Sinai Hospital (Toronto, Canada).
Outcomes	Expression levels of genes of interest were compared between samples from high grade bladder cancer and low grade bladder cancer.

Flow Cytometry

Plots

Confirm that:

☒ The axis labels state the marker and fluorochrome used (e.g. CD4-FITC).

☒ The axis scales are clearly visible. Include numbers along axes only for bottom left plot of group (a 'group' is an analysis of identical markers).

☐ All plots are contour plots with outliers or pseudocolor plots.

☒ A numerical value for number of cells or percentage (with statistics) is provided.

Methodology

Sample preparation	Cells were centrifuged and washed with PBS. The cell pellet was resuspendend in 1ml ice-cold PBS, and then 3ml of ice-cold ethanol was added. Cells were fixed at 4oC overnight. Fixed cells were centrifuged and washed twice in PBS and treated with RNase and resuspended in the wash buffer with propidium iodide (PI, 50µg/ml). Cells were anlyzed for PI content on a Gallios Flow Cytometer.
Instrument	Gallios Flow Cytometer 10 colors, 4 lasers from Beckman Coulter.
Software	ModFit LT v4.1 (Verity Software House)
Cell population abundance	Confluent cultures of the cell line MDA-MB-231 were treated or not with Media containing exosomes. Cells were harvested and counted before being processed for Flow Cytometry as described in sample preparation.
Gating strategy	a: Time vs PI Gate 1<br>b: PI integral linear vs PI peak linear (for removing doublets) Gate 2

☒ Tick this box to confirm that a figure exemplifying the gating strategy is provided in the Supplementary Information.
